# Toolkit for detecting misused epidemiological methods

**DOI:** 10.1186/s12940-021-00771-6

**Published:** 2021-08-19

**Authors:** Colin L. Soskolne, Shira Kramer, Juan Pablo Ramos-Bonilla, Daniele Mandrioli, Jennifer Sass, Michael Gochfeld, Carl F. Cranor, Shailesh Advani, Lisa A. Bero

**Affiliations:** 1 School of Public Health, University of Alberta, Edmonton, AB Canada; 2 Epidemiology International, Hunt Valley, MD USA; 3 Department of Civil and Environmental Engineering, Universidad de Los Andes, Bogotá, Colombia; 4 Cesare Maltoni Cancer Research Centre, Ramazzini Institute, Bologna, Italy; 5 Natural Resources Defense Council, Washington, DC USA; 6 George Washington University, Washington, DC USA; 7 Environmental and Occupational Health Sciences Institute, Rutgers Biomedical and Health Sciences, Newark, NJ USA; 8 Departments of Philosophy and Environmental Toxicology, University of California, Riverside, CA USA; 9 Terasaki Institute of Biomedical Innovation, Los Angeles, CA USA; 10 Georgetown University School of Medicine, Washington, DC USA; 11 Center for Bioethics and Humanities, University of Colorado Anschutz Medical Campus, Aurora, CO USA

**Keywords:** Disinformation, Ethics, Flawed science, Manufactured scientific controversy, Obfuscation, Partiality, Public policy, Research integrity, Scientific misconduct, Undue influence

## Abstract

**Background:**

Critical knowledge of what we know about health and disease, risk factors, causation, prevention, and treatment, derives from epidemiology. Unfortunately, its methods and language can be misused and improperly applied. A repertoire of methods, techniques, arguments, and tactics are used by some people to manipulate science, usually in the service of powerful interests, and particularly those with a financial stake related to toxic agents. Such interests work to foment uncertainty, cast doubt, and mislead decision makers by seeding confusion about cause-and-effect relating to population health. We have compiled a toolkit of the methods used by those whose interests are not aligned with the public health sciences. Professional epidemiologists, as well as those who rely on their work, will thereby be more readily equipped to detect bias and flaws resulting from financial conflict-of-interest, improper study design, data collection, analysis, or interpretation, bringing greater clarity—not only to the advancement of knowledge, but, more immediately, to policy debates.

**Methods:**

The summary of techniques used to manipulate epidemiological findings, compiled as part of the 2020 Position Statement of the *International Network for Epidemiology in Policy* (INEP) entitled *Conflict-of-Interest and Disclosure in Epidemiology*, has been expanded and further elucidated in this commentary.

**Results:**

Some level of uncertainty is inherent in science. However, corrupted and incomplete literature contributes to confuse, foment further uncertainty, and cast doubt about the evidence under consideration. Confusion delays scientific advancement and leads to the inability of policymakers to make changes that, if enacted, would—supported by the body of valid evidence—protect, maintain, and improve public health. An accessible toolkit is provided that brings attention to the misuse of the methods of epidemiology. Its usefulness is as a compendium of what those trained in epidemiology, as well as those reviewing epidemiological studies, should identify methodologically when assessing the transparency and validity of any epidemiological inquiry, evaluation, or argument. The problems resulting from financial conflicting interests and the misuse of scientific methods, in conjunction with the strategies that can be used to safeguard public health against them, apply not only to epidemiologists, but also to other public health professionals.

**Conclusions:**

This novel toolkit is for use in protecting the public. It is provided to assist public health professionals as gatekeepers of their respective specialty and subspecialty disciplines whose mission includes protecting, maintaining, and improving the public’s health. It is intended to serve our roles as educators, reviewers, and researchers.

## Background

Educated in the application of epidemiological methods, epidemiologists study where diseases occur, what causes them, and how to prevent them. According to *A Dictionary of Epidemiology* [1], the knowledge derived from epidemiological inquiry is not used solely for discovery purposes. It is also applied to control and prevent health problems and is used to restore, promote, and protect population health across all levels of society. Hence, by virtue of their focus on protecting the public’s health, epidemiologists, as a profession, are expected to serve the public, with the public interest trumping all others [2].

As an applied interventionist science, epidemiology is used not only to study health problems, but also to provide evidence to inform rational policy debate among interested stakeholders [3]. This evidence provides the scientific basis for correcting and, ideally, preventing health problems through government-driven health and social policy. Aside from informing policy, epidemiological data also provide the basis for individuals’ choices about lifestyle, diet, and other critical factors that influence health. Whether working as scholars, researchers, public health, or non-government agency professionals, as consultants, or even as expert witnesses in legal proceedings, the work-product and ultimate goal of the epidemiologist should be to promote and protect the public’s health, both at the population as well as the individual level.

Yet, in a world of conflicting interests, some parties may use the methods and language of epidemiology for personal gain or for corporate profit. They do so by manufacturing and casting doubt [4, 5] to confuse both policymakers and the public to the detriment of the public’s health. Goldberg and Vandenberg [6] have most recently identified commonly applied tactics used to misrepresent scientific discovery: spinning the facts to manufacture doubt, generating or perpetuating falsehoods. They point out that deceit can result in confusion that delays action by calling into question the scientific basis for concern.

Documents presenting best practices and ethics guidelines have been developed and adopted by the major epidemiology professional organizations to support the discipline and protect its integrity [7–9]. These provide the moral basis for epidemiology’s mission; they guide the normative practices of the discipline. While professionals who are not adherent to the guidelines can be called to account, there is no mechanism to ensure their implementation; moral suasion through peer pressure is the only enforcement mechanism.

In this commentary, our focus is on the discipline of epidemiology. The problems resulting from conflicting interests, and the strategies that can be used to protect public health from them, however, also apply to other public health disciplines, including risk assessment, toxicology, and exposure assessment.

## The role of undue influence in increasing uncertainty

Policy decisions are influenced by factors and inputs related not only to health risk assessments based on epidemiological data; they are also influenced by economic, political, social values, and special interest stakeholder considerations [10]. When policies informed by epidemiological evidence are debated in government, the preference is to make policy decisions in the presence of the greatest possible certainty. However, absolute certainty is not possible in science, given the inherent uncertainty that accompanies scientific inquiry. Consequently, epidemiologists are usually cautious and provide caveats for their findings. This creates an entry point for those bent on manipulating policy to promote confusion and engage in disinformation [11].

Poorly or inappropriately designed and executed epidemiological research that makes its way into the scientific literature serves to increase uncertainty. This renders the policy maker less likely to vote in favor of a policy change in support of public health. If the science can be muddied to foment uncertainty, or perhaps to mislead, a policy could ensue that leads to even more adverse population health risks.

Aware of this, a well-developed strategy among those with a vested self-interest in influencing and undermining policy, in a manner that is not consistent with the health of the public, is to find ways to increase scientific uncertainty, or to outright mislead. Science can be misused, either intentionally, through error, or from bias. In epidemiology, bias is defined as “an error in the conception and design of a study—or in the collection, analysis, interpretation, reporting, publication, or review of data—leading to results or conclusions that are systematically (as opposed to randomly) different from truth [1].”

Financial conflict-of-interest (COI), including author financial ties, review sponsorship, and journal funding, introduces a bias at all levels of the research and publication process [12]. Contrary to what many scholars might believe, this bias is not prevented by the peer review process [12]. Distortion and disinformation practices regarding scientific methods and evidence were intentionally employed by the lead industry in the early 1900s [13] and, since the 1950s, by the tobacco industry [14], and have since been honed by the asbestos industry [15–18]. The methods have become more sophisticated over time as played out from one industry to the next [19]. The goal is to pollute the scientific literature with studies designed to serve the interests of powerful sponsors and special interests. While scientists routinely disagree, the most intransigent disagreements arise when financial [20, 21], as well as political [22] interests are at play. When people become ill, die prematurely, and the health of future generations is placed in jeopardy, then transparency about the stakes involved becomes even more pressing.

Most recently, COVID-19 has taught us the importance of following epidemiological evidence in policy and health decision-making, especially in a global pandemic [3]. It has starkly revealed science’s politicization, corruption, and suppression [22]. Indeed, the pandemic has exposed relationships that need to be confronted if profiteering is to be contained, calling on values that support the public interest rather than self-serving relationships with industry [23]. Health harms are likely when the public is exposed to misinformation. Confusion ensues, which in turn creates a space for the mistrust of science, and the amplification of conspiracy theories through social media—resulting in aberrant behaviors that hamper vital public health measures [24].

Recognizing the range of factors affecting the policy process, and how they compete with one another, would help public health scientists appreciate the vulnerability of their discipline to being perverted for manipulating science, misguiding policy development, and supporting special interests. By following the money, one can identify the role that influence has played, and how this has encouraged the misuse of epidemiology [25]. The conduct of invalid science for generating “evidence” involving researchers financially supported by special interests (e.g., [26–29]), is a common and worrisome practice.

## Methods

Forces having direct or indirect financial stakes in policy interventions, especially those with a short-term focus on reports reflecting profits or personal gain to stakeholders, have been shown to be the most active in effectively working against the public’s health. Particularly, there has been a precipitous increase in the corporate funding of epidemiological research and an ever-growing reliance of academic institutions on such sources of funding. This has resulted in increasing instances of conflicting interests [30] which were brought to attention in the 2020 *International Network for Epidemiology in Policy* (INEP) Position Statement on *Conflict-of-Interest and Disclosure in Epidemiology* [31].

INEP is the major global network of epidemiologists with a focus on providing a bridge between epidemiological research and evidence-based, rational, government-formulated health policy that serves the public interest. It thus provides a unique forum to protect and promote public health, and works to ensure scientific integrity, promote ethical conduct in research, and support evidence-based research findings that are both independent and transparent. Its byline states: “Integrity, Equity, and Evidence in Policies Impacting Health.”

Thanks to investigative journalism, exposés of corporate and political influence in the United States (U.S.) in the period 2017–2021, reveal how the Environmental Protection Agency (EPA) under the Trump administration, the American Chemistry Council (ACC), and industry law firms colluded to weaken the EPA’s new chemical safety reviews [32]. The exposés also reveal how the fossil fuel industry has persisted over decades in influencing policy by obfuscating and denying negative impacts on human and planetary health [33–36]. Two seminal volumes, rich in well-established examples, were produced by the European Environment Agency [37, 38]. Furthermore, the harmful impacts of powerful influence through research sponsorship have been recently recognized, such that research and related professional sponsorship by Big Oil and Tobacco are being strongly discouraged [39].

On June 10, 2013, a few years prior to the aforementioned exposés, Dr. Margaret Chan, World Health Organization (WHO) Director General, made the following statement to the 8th Global Conference on Health Promotion, held in Helsinki, Finland: “*… In the view of WHO, the formulation of health policies must be protected from distortion by commercial or vested interests* [40].” INEP began to develop its Position Statement in 2014, soon after the WHO Director General’s pronouncement. What Dr. Chan noted indeed is an ongoing phenomenon.

With INEP working at the interface of research and policy, its mission includes recognizing and highlighting the misuse of data and potential corruption of the science practiced by epidemiologists. INEP comprises 24 national and international volunteer member associations and societies of epidemiology across five continents. It is registered as a 501(c)(3) public charity in the U.S. It is thus well positioned internationally to develop strategies to combat the misuse of epidemiological science. The INEP Position Statement [31] addresses two questions:

- How is it that public health policy remains under siege?
- Could public health be better protected through the improved management of *Conflict-of-Interest and Disclosure in Epidemiology?*

To address these questions, the INEP Position Statement [31] equips scientists with a set of tools to expose and root out so-called science that is designed to mislead and deceive. Hopefully, the actions of those drawing from the methods exposed in the Toolkit Table 1 (presented in the Results section below) to distort science should become less influential. Their influence will diminish because reviewers of epidemiological studies, be they peer reviewers or otherwise, should, by virtue of the toolkit, be more effective in identifying invalid science introduced to delay policy actions for protecting public health.

The toolkit’s role in the litigation process, from deposition to cross-examination in court proceedings, should also be helpful in both the pursuit of truth and for ensuring social justice. With the potential of the toolkit for bolstering the integrity of the discipline, we recognize that there are many journals with no or ineffective peer review; and, that industries have bought their own journals, limiting the extent to which the literature could be freed of corrupted science. The once-revered peer-review process is at risk, especially in journals controlled by vested interests.

Consolidation of the toolkit was thus included in the INEP Position Statement [31]. It is now made accessible as a standalone and expanded commentary. The compendium of tools provided in this commentary brings together work initially identified by Cranor [41, 42], and subsequently expanded upon by Soskolne [43–45] who saw the importance of expanding and consolidating this work to better arm epidemiologists, policymakers, and the scientific community with a greater appreciation for how epidemiological methods can be misused, abused, and perverted, counter to the advancement of knowledge and the public’s health.

This commentary has a role to play in not only bringing attention to, but also shining a light on, mechanisms of demonstrated influence and their harmful impacts on the advancement of science and the protection of public health. It therefore should be used as a teaching and training resource in graduate programs in epidemiology and other related public health disciplines. Every student emerging from any such program should be prepared to confront the world of malfeasance. Ideally, reviewers of manuscripts will be better positioned to separate public interest science from inappropriately designed studies that infiltrate the literature and hence the policy debate specifically to mislead science in the service of special interests.

## Results

As noted above, application of the epidemiological method can be influenced by interests that manipulate it in ways to produce findings that cast doubt, foment uncertainty, and seek to mislead decision makers. Unfortunately, some epidemiologists are susceptible to incentives that induce unprofessional conduct, thereby undermining the integrity of science [46–49]. The increasing reliance of public health institutions and epidemiologists on corporate funding, as well as the influence of politics on public health research, further exacerbate harms resulting from misusing the methods of the discipline and/or misinterpreting research findings.

To counter the types of forces noted above, a listing of key methods/techniques, arguments, and tactics has been assembled in the Table 1. It is provided to help identify how epidemiologists, usually financially supported by or under the influence of vested interests, manipulate, misuse, or inappropriately apply the methods of epidemiology, or misinterpret findings, to skew results and produce invalid science. The Table 1 is a toolkit that can be used as follows:

- By peer reviewers as a checklist of, or guide to key methodological parameters;
- To train epidemiologists and other healthcare professionals on the ways in which epidemiology can be distorted;
- To review the literature for invalid science or uninformative studies (e.g., underpowered studies, or misleading samples); and
- To identify who is misusing epidemiology.

The benchmark against which the toolkit can be compared is assembled from a selection of 12 foundational epidemiological textbooks, developed since the 1970s, with more recent editions cited here and used in epidemiology training programs [50–61]. This selection is somewhat arbitrary; any well-established textbook should suffice to gain understanding about the correct use of epidemiological methods.

Biostatistical methods are relied on for the design of specific epidemiological studies. As such, statistical methods are a critical component of the epidemiologist’s toolkit. Statistics is a discipline that has been in play for a longer period than what epidemiology has. It is not surprising, therefore, to find in the statistical literature articles extending over a longer timespan that bring attention to statistical mistakes that both researchers and practitioners can make in their work [62, 63].

Inappropriate techniques applied in epidemiology, including those that manipulate findings in ways that bias them toward the null, are assembled in the Table 1. These techniques may apply to the full realm of epidemiologic inquiry, including descriptive and analytical study designs. They include the use of unbalanced discussion that emphasizes findings not supported by the data, selective disclosure of competing interests, and publication in ‘pay-to-play’ journals without appropriate peer review, and with issues involving undisclosed conflicting interests.

**Table 1:** Toolkit of inappropriate applications of the epidemiological method

**--- Part A ---** **Epidemiology-specific methods/techniques used to foment uncertainty and cast doubt about cause-and-effect [through biased study designs and measurements producing invalid science]**			
**Item #**	**Method/technique**	**Effects**	**Reference(s)**
A1	Relying on statistical hypothesis testing; Using “statistical significance” at the 0.05 level of probability as a strict decision criterion to determine the interpretation of statistical results and drawing conclusions	Increases the probability of Type-II error; highly dependent upon sample size and statistical power; common strategy for dismissing study results that are indeterminate because of low power, or yield elevated risk ratios but do not reach an arbitrary level of statistical significance	[64, 65]
A2	Conducting statistically under-powered studies; ignoring Type-II errors	Sample size too small to detect an adverse effect, or adverse effect is too rare to be detected by a statistical study; asserting that a “negative” study (even if RR > 1) is proof of no effect. This can be addressed transparently by providing a power calculation Token studies are undertaken as a delay tactic because decision makers would rather not know the answer to a question, but want to give an appearance of concern. Thus, under-powered studies can arise through underfunding, which results in a shortfall in resources needed to be able to have the statistical power to detect a difference when one, in truth, exists. In addition, if not all needed information can be gathered to, for instance, properly control for confounding, the study will be of limited-to-no use. In such circumstances, what is actually being undertaken is a “token” study, not one that is capable of demonstrating an effect. For any number of reasons, epidemiologists may find themselves undertaking such a study. Token studies can serve special interests in two ways: (a) no effect will be able to be demonstrated, thus ensuring that the *status quo* is maintained; and (b) the special interest will be armed to say that a team of scientists is exploring a concern. This has the effect of being seen to be doing social good in addressing a health concern when, in fact, it will be a null study	[50, 62, 66, 67]
A3	Interpreting the statistical analysis or results inappropriately (see B8 below)	Concluding that a study with a risk ratio > 1 is “null” if it is not statistically significant at the 0.05 level; concluding that a risk ratio < 2 is a “null” result; concluding that lack of an elevated risk ratio is proof of no elevated risk (i.e., proof of the null hypothesis)	[62]
A4	Failing to use adequate follow-up methods	Not measuring appropriate endpoints through the pathogenesis of a disease process so that adverse effects can be identified (i.e., incomplete accrual problem)	[68]
A5	Failing to allow for adequate follow-up time	Not allowing sufficient time in a study for disease to manifest as with the latency between in utero exposure to diethylstilbestrol (DES) and appearance of cervical cancer of about 20 years, or the latency between exposure to asbestos and appearance of cancers of up to 45 years	[69–71]
A6	Introducing inappropriate representation of total person-years of exposure, seen especially in occupational health studies	Analyses based on a seemingly large number of person-years of exposure, which often represents short-term exposure among a large number of young workers in whom duration of exposure and latency are too short to observe an effect	[72]
A7	Contaminating controls	Control groups that include exposed persons (cohort studies) and early disease manifestation (case–control studies). It also includes placing controls into the exposed group, and exposed subjects/participants into control groups. For example, studies may use all employees as exposed, which includes unexposed office workers. And then they use nearby (fence line) residents as unexposed when this includes workers and fence line exposed residents	[73]
A8	Failing to statistically analyze or account for a broad range of exposure characteristics among the exposed group (cohort studies)	Potential dilution of effect of exposure-related risks by combining individuals with a broad range of exposure characteristics or histories without proper statistical adjustment or stratification	[74]
A9	Selecting inappropriate controls; failing to adhere to the requirement that controls should be representative of the population from which the exposed group (cohort studies) or the cases (case–control studies) emerged	Invalidates comparisons between study groups. A control group should be representative of the population from which the “exposed” group, or the case group, emerged. A good example is one comparing exposed workers in an industrial setting to the general population. Exposed workers in occupational studies are generally young and physically able to perform heavy jobs. They are not representative of the more biologically diverse general population. Such comparisons suffer from bias due to the well-established healthy worker effect. Thus, a general population control is not appropriate in an occupational study. A proper control group would be comprised of unexposed employees from the same industry with similar demographic characteristics	[75]
A10	Diluting / washing out / averaging effects in descriptive population comparisons	Combining all risk groups when it is in only a relatively small susceptible group in which the signal of effect will be demonstrated (that is akin to toxicology in which the correct strain of rodent is needed for demonstrating effects) Also, dividing the exposed population into so many exposure groups that each one fails to reach statistical significance (whereas an ‘ever-never’ comparison may be more appropriate) Also, using an ‘ever-never’ categorization to hide effects, when, in fact, it is the “peak exposed” workers that have the cancers (for example, formaldehyde for leukemia in the NCI studies)	[76]
A11	Ignoring known synergies among components of the mixture of chemicals	To study only a portion of a mixture to which people are exposed so as to dilute the risk of the whole, in which all components may not only interact to cause effects, but also work synergistically; to assess the risk of pesticide active ingredients individually, whereas commercial pesticide products contain multiple active ingredients plus adjuvants to enhance toxicity to the target species	[77, 78]
A12	Failing to account for the effects of exposure to complex mixtures in risk assessments	Virtually all exposures—be they ambient, occupational, or through other vectors—are complex mixtures. Analysis and representation of risk associated with one chemical or agent without taking into account the effects of the mixture may lead to distorted and erroneous risk estimates	[79]
A13	Using inadequate or insensitive laboratory methods, measurement practices, or instrumentation	If the criteria for a positive test or detection are stringent, then false positives will be reduced (high specificity), but false negatives will be increased (low sensitivity). Laboratory methods (which may include timing of sampling, storage conditions, etc.) and/or instrumentation that do not have adequate sensitivity or levels of detection will systematically underestimate exposure levels or effects; use of varying levels of detection in analyzing blood samples by different laboratory methods	[80, 81]
A14	Inappropriate analytical methods used in the statistical analysis	Failure to utilize appropriate statistical analytical techniques, as well as failure to adjust for confounding and/or effect-modifying variables, may lead to biased or inaccurate results. Examples also include analyzing matched case–control designs using methods that do not retain the matching This can shift the outcome in either direction resulting in false negatives or false positives. However, it is important to note that some of these will always shift to the null (like from small sample sizes, common cancer endpoints, etc.)	[71, 82]
A15	Suppressing data	Suppression bias from: Failure to include, in the statistical analysis, key findings in subgroups, or failure to report or publish the findings. Deliberate omission of findings or inappropriate groupings of outcomes to hide or dilute their impact. Omission of rare events from statistical analysis, or removing outliers could include removing peak exposures where all the cancers are to be found	[1, 20, 83, 84]
A16	Failing to recognize the validity of evidence from qualitative methods	The exclusive reliance on quantitative methods when qualitative research can provide both a context for the variables included in the quantitative analysis as well as a context for the interpretation of the quantitative findings	[85]
A17	Producing erroneous or biased meta-analyses and reporting them as representing a weight-of-evidence summary result	Meta-analysis includes studies with different study designs, or it selectively excludes studies that should have been included	[79]
A18	Using mortality instead of morbidity data for a cancer endpoint with a high survival rate	For example, using mortality instead of morbidity for breast cancer risk associated with ethylene oxide reduces the risk estimates	[51, 86]
**--- Part B ---** **Arguments used to delay action, maintain the *status quo*, and create divisions among scientists [imposing inappropriate standards and methods of suppression]**			
**Item #**	**Argument**	**Effects**	**Reference(s)**
B1	Insisting on the erroneous application of “criteria” for causation proposals (e.g., Bradford Hill viewpoints or aspects) in interpreting the weight of evidence in a causation analysis to infer causation	Guidelines in the form of “viewpoints” or “aspects” proffered for interpreting causation, including Bradford Hill, have been erroneously interpreted as required criteria, thereby leading to the dismissal of the weight of evidence that should properly be considered in health-protective policies. Despite outright errors in the Bradford Hill suggested guidelines, and his own expressed caveats about his proposed guidelines, the Bradford Hill guidelines are still cited by regulatory agencies, in legal proceedings, and by epidemiologists and healthcare professionals as a requirement for causation	[62, 68, 87]
B2	Failing to disclose a conflict-of-interest in the presence of a financial conflict-of-interest, financial control of agenda-driven funders, political influences, or vested interest goals (see C6 below)	The absence of objectivity / impartiality resulting in the application of a biased design or analysis, or selective interpretation of the findings	[10, 11]
B3	Ignoring mechanistic information suggestive of adverse effects	Ignoring or dismissing information pertaining to susceptible populations having increased risk so they can be studied rather than only studying the whole population; insistence on demonstrating a consistently elevated RR associated with a very rare outcome vs. showing elevated risk for broader classifications that are mechanistically related by virtue of similar biological activity	[88, 89]
B4	Exaggerating differences, or dismissing them, when toxicological studies suggest a potential human health hazard	Failure to synthesize knowledge from all disciplines that relate to a disease process	[82, 90]
B5	Ignoring related or families of molecular structures that predict potential health hazards	Insisting on the need for more research by ignoring prior knowledge or information on structurally related compounds (e.g., the perfluorinated alkylate substances [PFASs])	[91, 92]
B6	Focusing on studying and reporting only general population effects to the detriment of identifying and protecting from adverse health impacts the most vulnerable, chemically sensitive, and genetically susceptible in society, including children and pregnant women	Failure to protect vulnerable sub-populations and failure to recognize heightened susceptibilities; e.g., of the developing brain to neurotoxicants, or from heightened risks to immune-compromised persons; erroneously assuming that a lack of data in the literature about sub-populations or about rare conditions indicates no risk of disease associated with exposure Neurotoxic chemicals tend to be more harmful when exposures take place during fetal and early life development, as recognized by scientists, but not by regulatory agencies	[75, 83]
B7	Demanding an unusually high degree of certainty for the public health problems to be addressed; claims that more data are needed for “proof” of elevated risks; rejection of the Precautionary Principle	Demanding proof “beyond a reasonable doubt,” typical of criminal law proof requirements, although risk of a health hazard may vary due to differential susceptibility and may not be discernable beyond a reasonable doubt for an individual. In U.S. tort litigation, the typical standard of proof is “preponderance of the evidence” or “balance of probabilities” that require a determination of “more probable than not.” Environmental health advocates and public health scientists endorse a lower level of probabilistic evidence, whereas industry argues for higher standards of proof. In summary, public interest groups err on the side of caution to protect public health, whereas polluting industries press for an unattainable standard of proof that science cannot often meet. Thus, the appropriate application of science and weight of evidence support are not used in judgments as to public health policies or litigation outcomes	[13, 14, 18]
B8	Demanding that any observed odds ratio / relative risk between exposure and disease must be 2 or greater before the study can be admitted to support expert testimony [See A3 above]	The odds ratio for a population may be 1.5 and represent millions of people at risk in a large population—as in pesticide exposure and autism—leading to a larger public health impact. This demand fails to recognize the public health importance of population attributable risk for prevalent exposures; while risk estimates may be low, the absolute number of affected people can be large. In the example provided, it is difficult to get an OR higher than about 3 because of all the practical issues of conducting a study such as multiple exposures, intermittent (seasonal) exposures, undocumented exposures due to undocumented workers not wanting to report, etc	[93–95]
**--- Part C ---** **Tactics invoked to misdirect policy priorities through influence [imposing undisclosed values from the positions taken by special interests]**			
**Item #**	**Tactic**	**Effects**	**Reference(s)**
C1	Assuming that “no data” equates to “no risk”	Lack of research about a public health issue—and a paucity of data—does not equate to “no risk.” However, the absence of data (because of the failure to conduct studies) is often invoked or misinterpreted as evidence of no risk. The absence of scientific research, including the absence of epidemiological research, does not equate to “no risk.” Mechanistic and toxicological data can be sufficient evidence to indicate human risk	[7, 64, 84, 96, 97]
C2	Failing to study a critical public health issue because of political influence, financial interests, or influence of special interest groups resulting in a Repression Bias. We should not lose sight of the fact that some studies are never done because approval for them was, for some reason, not granted. Sometimes the reason is because the topic is repressed	Critical public health threats, including climate change, firearm violence, obesity/diet, and others have not been properly addressed due to the improper influence of special interests. Repression Bias arises in situations in which a line of inquiry is not pursued because the researcher is, consciously or subconsciously, aware that pursuing such a research question would upset the dominant culture/paradigm, or the funding agency. The research question may never be investigated because funding is not made available from the funding agency for its study. In practice, students may be directed away from such questions if the funding support needed to complete the research component of their graduate program cannot be secured. In the absence of new information, no action is demanded of those bearing responsibility. Students and researchers persisting in researching that which may offend the establishment could find themselves unemployable or unemployed, respectively	[1, 11, 15, 84, 98]
C3	Failing to generalize health risks, and restricting the assignment of risk to local populations of exposed people despite demonstrated effects in humans elsewhere	Refusing to accept that health effects observed in one exposed population are likely to operate in much the same way in a similarly exposed population in a different location	[14, 15]
C4	Neglecting to apply or dismissing the Precautionary Principle when there is evidence to justify interventions to reduce/eliminate exposures	Insistence on occurrence of dire public health impacts (e.g., significantly increased morbidity or mortality rates) before action is taken although the weight of the evidence supports excess risk of adverse effects from exposure	[15, 35, 36]
C5	Failing to be transparent in making explicit those value judgments that underlie decisions about selecting appropriate standards of evidence to draw policy-relevant conclusions (i.e., in suppressing dominant interests and values)	Failing to discern acceptable risks as a policy determination vs. the actual risk of exposure	[7, 8, 28]
C6	Infiltrating editorial boards, scientific review panels, and decision-making bodies of all kinds (see B2 above)	By gaining a presence, the ability of impartial representatives to influence decisions and ensure a voting majority to support a particular stakeholder’s vested interest that is not consistent with that of the public interest	[99, 100]
C7	Misdirecting policy priorities through influence	Influencing the research agenda by funding research that supports a stakeholder industry’s position OR detracts from harm of their product. This is important because it is not about influencing individual studies, but rather the entire evidence-base relevant to a policy. The review includes a number of examples: for instance, tobacco industry funding research on contaminants of indoor air OTHER than second-hand smoke; and, sweetened beverage industry funding research on the benefits of exercise rather than on the harms of sugar	[101]

In brief, the above Table 1, constituting the toolkit, is organized in three parts:

Part A of the Table 1 reflects on how the findings from epidemiological inquiry are affected by the design of studies, as well as on the how and what is being measured. We have compiled epidemiology-specific methods/techniques used to foment uncertainty and cast doubt about cause-and-effect through biased study designs and measurements producing invalid science.

Part B of the Table 1 reveals arguments that impose inappropriate standards and methods of suppression counter to the principle of openness and transparency. We have compiled arguments used to delay action, maintain the status quo, and create divisions among scientists by imposing inappropriate standards and methods of suppression.

Part C of the Table 1 identifies tactics imposed by those serving special interests to upset the very foundation of reason as it pertains to the core values and methods of the discipline. We have compiled tactics invoked to misdirect policy priorities through influence imposing undisclosed values from the positions taken by special interests.

## Discussion

Since the compilation of this toolkit, the literature has, over the past year, seen many more examples of conflicting interests and failures to disclose them. Each example exposes the inappropriate role of influence-wielding at all levels of scientific inquiry and knowledge advancement.

In this commentary, we focus on the toolkit aspect of the INEP Position Statement [31], and thus limit ourselves in this discussion to one recent contribution to the topic of bias assessment because of its focus on methods. It appears in a 2020 commentary by Steenland et al. [102] in which they consider risk of bias (RoB) assessments and evidence syntheses for observational epidemiological studies of environmental and occupational exposures. RoB tools are used to evaluate epidemiological studies as part of evidence synthesis, the latter requiring a broader approach than simply evaluating RoB in individual studies. Those authors recognize the need to include classical considerations for judging causality in human studies, “as well as triangulation and integration of animal and mechanistic data.”

As with the INEP Position Statement [31], Steenland et al. [102] recognize conflict-of-interest, which can create the potential for bias, a bias that is not always assessed in RoB tools. They point to strong evidence that “studies authored by those with vested interest are generally favorable to those interests, hence the need to disclose potential conflict of interests.” In the view of Steenland et al. [102], if specific biases are present, reviewers should be able to detect them in evaluating studies. However, “generally not included in current risk of bias tools is potential bias because of problems in statistical methods. Concerns include choice of an inappropriate and badly fitting model, failure to model exposure–response or to evaluate different exposure–response models, incorrect use of mixed models, incorrect use of Bayesian techniques, violation of statistical assumptions (e.g., normal residuals in linear regression), overadjustment for covariates related to exposure but not to outcome, adjusting for causal intermediates, etc.”

We note that statistical models and methods are quite complex. As such, many epidemiologists and peer reviewers, as well as the general reader, may not be able to evaluate their appropriateness. Yet, bias due to COI has been increasingly considered and assessed in systematic review methodologies and RoB tools of epidemiological studies, including the Navigation Guide [103], and the WHO/ILO Joint Estimates of the Work-related Burden of Disease and Injury [104].

Another domain of evidence synthesis that does not entail bias per se is “informativeness.” Consideration in this domain includes whether the study has a large enough sample size, whether the study has sufficient latency, whether results have been reported selectively, and whether the study has sufficient exposure contrast to see an effect of exposure on outcomes. This domain is sometimes called sensitivity in some evidence syntheses.

There is considerable overlap between the strategies identified in the toolkit of Goldberg and Vandenberg [6] and those independently identified in our Table 1 (above). This lends credence to our respective approaches for addressing the challenge of manufactured doubt. It adds a degree of validation to each of our respective Tables revealing strategies, arguments, and tactics used in doubt mongering. In the clinical realm, regarding disclosure as a mechanism for mitigating the effects of COI, Rimmer [105] notes that, until the introduction of a mandatory register of doctors’ interests, patients would have no idea who was funding their doctor’s voice, or who might be biased towards certain treatments. Related health professional bodies are thus calling out the biases to health and science induced by commercial interests.

In practice, broad opportunity exists to publish invalid science owing to: (1) the existence of predatory pay-to-play journals; (2) open access journals with little peer review; and (3) editors/peer reviewers who themselves have a COI and/or little-to-no knowledge of the topic under review. Given this, those who rely on the published literature, in both government and among the public, including the media, should be aware that research strategies exist that can be misleading. Above all, since professional epidemiologists are the gatekeepers of the discipline, they have the moral responsibility to execute its mission. It therefore behooves them, along with other healthcare professionals, to be familiar with this toolkit as but one mechanism for better ensuring the maintenance of professional standards of integrity [43] throughout the public health sciences.

## Recommendations

Common practices to distort and misapply epidemiological science should be recognized and called out professionally when they occur. INEP member organizations, academic institutions, and other public health professionals can adopt INEP recommendations and strategies for COI management that include identification, avoidance, disclosure, and recusal [31]. It would be of added benefit to incorporate this commentary into the curriculum of graduate training programs in the health sciences and in medical schools to equip entry-level professionals to better serve as gatekeepers of the discipline.

The toolkit can be used as a guide in what to look for, to train epidemiologists and others on how epidemiology can be distorted, to evaluate the literature for invalid science or uninformative studies (e.g., underpowered studies), and to identify who it is that is misusing epidemiology along with their motivations. It can be used as a checklist for critically appraising descriptive or analytical studies pre- and post-publication, policies, and arguments in legal proceedings.

In summary, techniques to manufacture and cast doubt (i.e., irrational skepticism), targeted at policymakers and consumers through the misapplication of the epidemiological method, claim that:

- The science is unclear
- There is dissent (where the evidence is clear)
- The data are inconclusive
- Scientists are biased—“You can’t trust scientists”
- Regulation is unjustified—“It’s a slippery slope.”

This is achieved through:

- Delaying action
- Influencing policy decisions—risk factors for bias
  - ◦ Pulls: Vested interest (stand to gain personally)
  - ◦ Pushes: Lobbying.

Defenses that work against epidemiology being misapplied include:

- Correctly applying and clarifying the methods of statistical inference
- Exposing undisclosed COI
- Recognizing erroneous and misleading interpretations of underpowered studies
- Acknowledging the scientific assessment of uncertainty
  - ◦ Bias; statistical (aleatoric) uncertainty; epistemic uncertainty
  - ◦ Model uncertainty; parameter uncertainty
  - ◦ Expected value = (value of outcome) X (probability of outcome)
  - ◦ Uncertainty intervals
- Highlighting when the logic of an argument is invalid
  - ◦ False premises
  - ◦ Invalid argument
  - ◦ Misapply conclusions
- Exposing the motives of researchers, journal editors, peer reviewers, decision makers and other stakeholders in the policy process
- Critically appraising the evidence as presented
- Publishing standards for good practice, e.g., the INEP Position Statement
- Calling out malpractice.

While the Council on Publication Ethics (COPE) has guidelines designed to keep the literature free of corrupted or poor science, they are known to be inadequately enforced and are insufficient to stop the manipulation of the literature [16, 106, 107]. Actions on the part of the epidemiology community, as well as the broader health sciences, could help to change this as the problems are recognized and addressed. The scientific community should engage by recognizing and professionally calling out common practices used to distort and misapply epidemiological and other health-related sciences.

To demonstrate the seriousness of serving as gatekeepers with the moral responsibility to uphold professional standards, epidemiologists could expand upon the INEP Position Statement, using it as a launching pad to write other documents (e.g., other position statements, policy briefs, commentaries, letters, case studies, and editorials) to extend the reach of INEP’s Position Statement. Ultimately, exposing the public and policymakers to the INEP Position Statement will provide reassurance about the seriousness that professionals hold in protecting the public’s health. It is possible that, in return, with enhanced credibility in the profession, funding could be made available to support organizations like INEP as valued counterweights to the manipulation of this key public health science whose mission it is to serve the public interest above any other.

Epidemiologists and other health professionals must not be naïve. They need to remain vigilant to the variety of forces at play that influence both science and policy. In addition to vigilance, personal integrity is required to counter the influence of economically powerful entities and corrupt and/or morally bankrupt governments whose focus is not on protecting public health, but rather on protecting narrow, special interests.

This said, there are frailties in both human beings as well as in governmental structures. Sensitive to this reality, we provide specific short-term objectives that each epidemiologist could immediately implement: Recognize our professional obligation to be vigilant and especially careful in peer review to avoid contaminating the literature with invalid or poor science; and, support added oversight, as in Human Research Ethics Boards (HREBs) or Institutional Review Boards (IRBs), on the need to keep ourselves on track with the moral responsibility for being aware of and compliant with our profession’s ethics guidelines.

We recommend accepting that uncertainty is inherent in science. In our role as scientists, we strive to be value-neutral or value-free, but the human instrument is, in fact, incapable of achieving this point of neutrality or impartiality. Consequently, we need to look first to ourselves, because causal inference is a function of who it is that is making the inference which, in turn, is a function of how we apply our scientific methods. Anything that we can do to build protections into the system of self-governance that is expected of professions like epidemiology, we ought to engage with and embrace.

## Conclusions

This novel toolkit exposes the negative impacts of the misuses of epidemiology. As such, it provides an essential foundation for expanding the science and methods of argumentation (i.e., disagreement) through formal logic and dialectics. While beyond the scope of this commentary, the challenge posed to develop an application (i.e., an app) based on the Table 1—to more efficiently review the literature and for rooting out invalid science and misleading conclusions—warrants further exploration in this philosophical context.

The toolkit, consistent with INEP’s mission, is made available to protect the public. It is provided to assist public health professionals whose mission includes protecting, maintaining, and improving the public’s health. Its utility lies in our more specific roles as educators, reviewers, and researchers. It is to be used to detect and professionally expose the misuse and distortions of epidemiology that result in misinformation that contaminates the literature, a domain on which the advancement of science and public policy rely.

## Data Availability

DOIs and hyperlinks are included throughout the literature cited.

## Abbreviations

ACC: American Chemistry Council
INEP: International Network for Epidemiology in Policy
COI: Conflict-of-Interest/conflicting interests
COPE: Council on Publication Ethics
EPA: United States’ Environmental Protection Agency
HREB: Human Research Ethics Board
IARC: International Agency for Research on Cancer
IRB: Institutional Review Board
OR: Odds Ratio
RoB: Risk of bias
RR: Relative Risk
U.S.: United States
WHO: World Health Organization
