# Correction: Toolkit for detecting misused epidemiological methods

**DOI:** 10.1186/s12940-022-00938-9

**Published:** 2022-11-12

**Authors:** Colin L. Soskolne, Shira Kramer, Juan Pablo Ramos-Bonilla, Daniele Mandrioli, Jennifer Sass, Michael Gochfeld, Carl F. Cranor, Shailesh Advani, Lisa A. Bero

**Affiliations:** 1 School of Public Health, University of Alberta, Edmonton, AB Canada; 2 Epidemiology International, Hunt Valley, MD USA; 3 Department of Civil and Environmental Engineering, Universidad de Los Andes, Bogotá, Colombia; 4 Cesare Maltoni Cancer Research Centre, Ramazzini Institute, Bologna, Italy; 5 Natural Resources Defense Council, Washington, DC USA; 6 George Washington University, Washington, DC USA; 7 Environmental and Occupational Health Sciences Institute, Rutgers Biomedical and Health Sciences, Newark, NJ USA; 8 Departments of Philosophy and Environmental Toxicology, University of California, Riverside, CA USA; 9 Terasaki Institute of Biomedical Innovation, Los Angeles, CA USA; 10 Georgetown University School of Medicine, Washington, DC USA; 11 Center for Bioethics and Humanities, University of Colorado Anschutz Medical Campus, Aurora, CO USA

**Correction: Environ Health 20, 90 (2021)**

**https://doi.org/10.1186/s12940-021-00771-6**

Following publication of their original article [1], the authors identified an error in their declaration of Competing Interests and ask that what was previously indicated as “…no competing interests” be replaced with the following ERRATUM:

ERRATUM:

The BMC Competing Interests Policy states: “…A competing interest exists when the authors’ interpretation of data or presentation of information may be influenced by, or may be perceived to be influenced by, their personal or financial relationship with other people or organizations.” At the time of publication in 2021, our team of nine co-authors interpreted the BMC Policy as not requiring declaration of any real or perceived competing interest. Since then, however, the authors decided that any past role for which professional activities or payments were involved ought to have been stated, for the sake of greater transparency. We submit the following as an ERRATUM under the topic of Competing Interests:

S. A., L. A. B., and D.M., three of the team of nine authors, declare that they had, and continue to have, no competing financial or non-financial interests.

The remaining six co-authors declare as follows:

C. L. S. is retired and unfunded since 2013. In the recent past, he served, for about three years prior to 2013, as an expert witness in asbestos tort actions and a diacetyl case on behalf of plaintiffs, the monies from which went into a University of Alberta-managed research account. As a professional legacy, he bankrolled the International Network for Epidemiology in Policy (INEP) (2011–2016) as a voluntary professional society in the hope that it might become self-sustaining in the pursuit of science in the public interest. He has endorsed amicus briefs in support of plaintiffs in asbestos litigation. His doctoral thesis, in the early 1980s, was funded by the Exxon Corporation.

S. K. has no financial competing interests. She was the Founder and CEO of Epidemiology International. She has served, until 2016, as an expert witness in toxic tort and pharmaceutical tort litigation on behalf of plaintiffs. Payment for such work was made to her company, Epidemiology International. Epidemiology International has also performed work under contract for industry, pharmaceutical companies, the U.S. federal and state governments, and patient advocacy groups.

J.P.R.-B. was invited, between 2015 and 2019, ad honorem, to provide his expert opinion in the Colombian Senate in support of a national asbestos ban that had been proposed, a ban that was finally approved in 2019.

J. B. S. is employed by the Natural Resources Defense Council, a U.S.-based environmental non-profit group that engages in public advocacy, lobbying, and litigation to expand protections for the environment and public health and to enforce existing environmental laws regulating toxic chemicals, including some of the chemicals identified in this manuscript. This work receives partial funding from Deer Creek Foundation, and Passport Foundation.

M. G. retired in 2014 and has not done paid consulting work on any environmentally related issue since that time. His last paid consulting work was prior to about 2010. He wrote scientific papers and served as an expert on hexavalent chromium clean-up and was deposed by the principal responsible parties fighting a state-ordered cleanup of major hexavalent chromium contamination. The research and deposition were part of his academic job description, and no fees were paid directly to him.

C.F.C. has, from time to time, been sought after to serve on science advisory panels and governmental agencies to emphasize issues concerning science in the law. He has participated in various amicus briefs with no compensation. From about 2009, he wrote reports and consulted on scientific reasoning for a few law firms for pay, but he did not testify except in one Daubert hearing before a judge.

Our collective goal is transparency in the pursuit of truth through the advancement of science in the public interest.
